# Editorial: 3Rs approach (replace, reduce and refine animal models) to improve preclinical research

**DOI:** 10.3389/fphys.2022.1040575

**Published:** 2022-10-06

**Authors:** Laura Calvillo

**Affiliations:** Istituto Auxologico Italiano IRCCS, Milan, Italy

**Keywords:** laboratory animal, husbandry, organoid cell culture, analgesic, bioreactors, genotying, breeding colonies

“3Rs” means *Replacement, Reduction, Refinement*, a conception developed more than 50 years ago to improve welfare of animals used in research. This concept should be widely known by biomedical researchers, being a fundamental principle of investigations involving laboratory animals. It means that when experiments with laboratory animals are necessary, scientists should consider if a possible animal-free model can be used. If not, they should apply proper statistics and methods to Reduce the number of animals without losing the scientific quality of the work and should improve the procedures to Refine the animal model, in order to reduce unnecessary suffering or stress. It is known that pain and stress can affect homeostasis creating suffering in animals and increasing the risk of bias. Also, many relevant questions cannot be addressed only with use of laboratory animals or cell cultures on a Petri dish. A 3Rs approach to preclinical investigations ensures that the results of the research are of high quality, increasing the translational value of the data itself and encourages the development of technologically advanced models. In this Research Topic, all the three aspects of Replacement, Reduction and Refinement were presented by the various authors, providing important tools to researchers working in basic research. The paper of Vitale and Ricceri presents an important overview of the Research Topic, emphasizing the potential issue troubling a correct application of 3Rs, like the so-called “methodological inertia,” as well as the great importance of continuing to spread the “3Rs” principles. It presents a brief but complete assessment of the aspects related to each “R,” and useful and practical links and tables.

The experimental work of Durst et al. addresses the problem of pain management in a model of pancreatitis in mice, analysing the effect of several analgesic treatments administered in drinking water, thus describing a Refinement method. A complete series of behavioural, histological and biochemical tests are described, providing important procedures for those researchers using the same animal model. The article is an important example of the application of statistics and international guidelines to studies where procedures can have an important impact on laboratory animals’ welfare.

The contribution of VanDenBerg et al. deals with the problem of genotyping mice, a procedure where a continuous update on the Refinement is essential. They compare three methods to genotype and breed colonies on a large scale, providing interesting information for typical husbandry procedures in both academic environment and for an industrial context.

The other two contributions to this Research Topic address the topic of Replacement. The review of Cacciamali et al. provides an overview of the tools currently developed by bioengineering, which promise to be able to Reduce, if not even in some cases Replace, the laboratory animal. Authors describe the difference between cells cultivated in 2D or in 3D (organoids) and present the various experimental situations where the different devices have proved useful. They also describe the problems not yet solved and the current limitations on being able to completely Replace the animal.

The experimental work of Barra et al. describes a very interesting dynamic *in vitro* model of the blood–brain barrier (BBB), developed by using the IVTech millifluidic bioreactor systems. This model allowed authors to create a more physiological environment by ensuring a circulation of the medium and nutrients, thanks to the continuous laminar flow. With this advanced *in-vitro* BBB, they demonstrated that liposomes loaded with specific molecules efficiently crossed the barrier, describing a new possible Replacement model with great potentiality in pharmacological studies as well as in neuroscience. The goal of this Research Topic was to bring together the most recent and advanced work on the 3R’s methods in order to share information that can help spread a 3R’s approach to preclinical research. A collection of papers describing advances in reducing pain and stress in laboratory animals as well as studies on next generation *in-vitro* tools were produced by the Authors, who contributed with their work to achieve the scope of this Research Topic.

